# Enrichment of type 1 innate lymphoid cells in the course of human atherosclerotic plaque development suggests contribution to atherogenesis

**DOI:** 10.3389/fimmu.2024.1354617

**Published:** 2024-04-04

**Authors:** Kartika R. Pertiwi, Marcel B. M. Teunissen, Gabrielle Krebbers, Martine C.M. Willems, Laurens Huisman, Cindy Poelen, Allard C. van der Wal, Onno J. de Boer

**Affiliations:** ^1^ Department of Pathology, Amsterdam University Medical Centers, location Academic Medical Center, University of Amsterdam, Amsterdam, Netherlands; ^2^ Faculty of Medicine and Department of Biology Education, Faculty of Mathematics and Natural Science, Universitas Negeri Yogyakarta, Yogyakarta, Indonesia; ^3^ Department of Dermatology, Amsterdam University Medical Centers, location Academic Medical Center, University of Amsterdam, Amsterdam, Netherlands; ^4^ Department of Vascular Surgery, Amsterdam University Medical Centers, location Academic Medical Center, University of Amsterdam, Amsterdam, Netherlands; ^5^ Department of Vascular Surgery, Flevoziekenhuis, Almere, Netherlands

**Keywords:** innate lymphoid cells, atherosclerosis, innate immunity, inflammation, lymphocytes, immunohistochemistry

## Abstract

**Introduction:**

Innate lymphoid cells (ILCs) have been implicated in multiple pathologic conditions, including atherogenesis, as documented in experimental mice studies, however, their role in atherosclerosis in humans remains unexplored.

**Methods:**

Here, we identify ILCs and their dynamics in early, advanced, and complicated human carotid- and aortic atherosclerotic plaques, using a multiplex immunohistochemical quadruple-staining technique with prototypic transcription factors T-bet, GATA3, or RORgt for identification of the ILC1, ILC2 and ILC3 subsets, respectively, in combination with lineage markers CD3, CD20/ CD79a and CD56 to exclude other lymphoid cell types. ILC subsets were quantified, and to put this in perspective, their numbers were expressed as percentage of the total number of infiltrated lymphoid cells and related to the frequency of conventional T cells, B cells, NK cells, and NKT cells.

**Results:**

All ILC subsets were present in every different stage of atherogenesis. ILC1s were the most abundant ILC subset, and their numbers significantly increased in the course of plaque development, but paradoxically, their relative frequency was reduced because of a higher increment of T cells and B cells. The numbers of ILC2s and ILC3s also gradually increased, but this trend did not achieve significance. T cell subsets always significantly outnumbered their ILC counterparts, except for the early lesions where the proportion of ILC1s was markedly higher, albeit not significant.

**Discussion:**

The high abundance of ILC1s in the early stages and further significant enrichment in later stages, suggest they may participate in the initiation and development of atherogenesis, and thus, may represent a novel target to prevent or treat atherosclerosis.

## Introduction

Atherosclerosis, the underlying cause of acute myocardial infarction, stroke, and peripheral artery disease, is a chronic inflammatory disease of the large and middle-sized arteries (1, 2). Extensive studies on human plaques, as well as experimental animal models, have shown that both innate and adaptive immune responses contribute to pathogenesis of atherosclerosis (1–3). The earliest stage of atherosclerosis is triggered by innate immune responses directed against modified lipoproteins (2, 4). In later stages of the disease, specific, adaptive immune responses are believed to become important (1–3). In the last 30 years, immunohistochemical studies have extensively characterized the composition and nature of the innate and adaptive inflammatory infiltrates in human atherosclerotic tissue. Macrophages and T lymphocytes are the most abundant inflammatory cell types in human atherosclerotic plaques (5–8).

Innate lymphoid cells (ILCs) are a distinct family of lymphocytes, which are derived from common lymphoid precursors. Unlike T cells and B cells, ILCs lack rearranged antigen-specific receptors, and consequently, are unable to respond in an antigen-specific manner (9–11). Instead, they respond to various microenvironmental signals (e.g. cytokines and alarmins) that are released by the surrounding tissue (9–11). Most ILCs reside in tissues with a barrier function, like the skin (12) and the mucosal surfaces of the intestine (13) and lung (14), whereas in the blood circulation their frequency is very low (15, 16). ILCs are multifunctional and involved in protective immune responses against microorganisms, lymphoid tissue formation, but also in tissue homeostasis and tissue remodeling after damage (9–11).

At present, five distinct subsets of ILCs are recognized: cytotoxic natural killer (NK) cells, lymphoid tissue inducer (LTi) cells, and the helper subsets, ILC1s, ILC2s and ILC3s (11). ILC1s, like NK cells, require transcription factor (TF) T-bet for their development and function, and produce the pro-inflammatory cytokine IFN-γ (17). ILC2s require TF GATA3 and secrete cytokines IL-4, IL-5 and IL-13 under the influence of IL-25 and IL-33 (15). ILC3s depend on the TF RORγt and produce IL-17 and IL-22 in response to IL-1 and IL-23 (18). The ILC1, ILC2 and ILC3 subset-defining TFs and hallmark cytokines resemble those of the polarized adaptive CD4^+^T helper (Th) cell subsets Th1, Th2 and Th17, respectively, and in addition, CD8^+^ cytotoxic T (Tc) cells may also express T-bet, GATA3, and RORγt and can be designated as Tc1, Tc2, Tc17 cells, respectively (19).

Information on a possible contribution of ILCs in the pathogenesis of atherosclerosis is limited. In a mouse model of atherosclerosis, it was shown that ILC1s are the dominant ILC subset in atherosclerotic plaques and involved in reinforcing plaque formation (20). ILC2s are also present in atherosclerotic tissue in mice, however, they are not promoting atherogenesis but rather have a regulatory function attenuating atherosclerosis (21–24).

At present, it is unknown whether ILCs are present in human atherosclerotic plaques or may play a role in atherogenesis. Recently, we have developed a quadruple-color multiplex immunohistochemical staining protocol for detection of ILC1, ILC2 and ILC3 subsets in formalin-fixed paraffin-embedded specimens, using subset-associated archetypical TFs T-bet, GATA3, and RORγt as positive identifiers in combination with lymphoid lineage markers to exclude non-ILCs (25). Using this quadruple staining approach, we have identified and quantified ILC1s, ILC2s and ILC3s in human atherosclerotic lesions *in situ* in all stages of atherosclerotic plaque development, from very early to advanced and complicated (ruptured) plaques. Furthermore, this staining approach enabled the concomitant determination of the 3 types of T cells (Th1/Tc1, Th2/Tc2 and Th17/Tc17 cells, which will be further designated as T1, T2, and T17 cells, respectively), putting the frequencies of ILC subsets into perspective of the proportion of intralesional T lymphocytes.

## Materials and methods

### Specimen selection and classifications

Atherosclerotic plaques (n=65, obtained from 47 patients) were retrieved from the archives of the Department of Pathology, Amsterdam University Medical Centers (location AMC), Amsterdam, the Netherlands. Specimens included carotid endarterectomies (n=35) and aortic specimens (n=12) which were obtained during surgery. Plaques were directly fixed in formalin for 24–48 hours, decalcified in 10% EDTA pH 7.4 for at least 3 weeks, and subsequently embedded in paraffin and stored as tissue blocks. Sections (5 µm) of each specimen were cut, and the first section was histologically stained with hematoxylin and eosin (HE) to determine the plaque stage according to the American Heart Association classification of atherosclerosis (26). These groups were further graded in 3 categories as either early (diffuse intimal thickening, fatty streak or pathologic intimal thickening), advanced (lipid-rich regions, fibrous-calcified plaques or a combination of these features) or complicated (hemorrhage, erosion or rupture). Finally, 28 early, 23 advanced and 14 complicated lesions were identified and selected for further analysis.

All specimens were leftover materials from clinical interventional procedures and were anonymously used. The use of these specimens was approved by the Committee Review Biobanks (CTB.2015-081). Retrospective collection was conducted in accordance with ethical guidelines of Amsterdam UMC Research Code, based on the ‘Code of conduct Health Research’ (Dutch Committee on Regulation of Health Research).

### Immunohistochemistry

Identification of ILC and T cell subsets in human plaques was performed using a multiplex quadruple staining method as previously described (25). In short, after deparaffinization in xylol, rehydration in graded alcohols, quenching of endogenous peroxidase with methanol/H_2_O_2_ and heat induced epitope retrieval in Tris-EDTA, pH 9.0 at 98°C for 20 minutes, sections (5 µm thickness) were stained using appropriate dilutions of either rabbit anti-human T-bet (clone EPR9302; Abcam, Cambridge, UK), rabbit anti-human GATA3 (clone EPR16651; Abcam) or mouse anti-human RORγt (clone 6F3.1; Sigma-Aldrich Chemie, Zwijndrecht, Netherlands). Then, the sections were incubated with horseradish peroxidase-conjugated anti-mouse/rabbit BrightVision (Immunologic, Duiven, the Netherlands). After enzyme detection with NovaRed (Vector Laboratories, Burlingame, CA), counterstaining with hematoxylin and mounting with gelatin/glycerol, sections were digitized using a Philips Ultra-Fast Scanner (Philips Digital Pathology Solutions, Best, the Netherlands). Subsequently, the sections were prepared for an additional staining round by removing the coverslip in warm tap water. Stained sections were stripped from dyes and immune-complexes in Tris-SDS with 0.7% β-mercaptoethanol (50°C for 30 minutes) and washed in running tap water. The staining, digital acquisition, and stripping cycle was repeated three times successively, using antibodies against CD56 (clone MRQ-42; Cell Marque, Rocklin, CA) to detect NK cells, CD3 (clone A-1; Santa Cruz Biotechnology, Heidelberg, Germany) to detect T cells, and a mixture of CD20 and CD79α (clone L26 from Immunologic and clone JCB117 from Dako, Glostrup, Denmark, respectively) to detect B cells and plasma cells. Positive and negative control sections with human tonsil tissue were always included. The order of markers to be detected was determined in detail in our previous study, and was based on the degree of epitope loss during the cycles of staining/destaining (25).

In order to detect T-bet expression by intraplaque macrophages, we applied a standard double staining technique, using rabbit anti-human T-bet (clone EPR9302) and mouse anti-CD68 (clone PGM-1; Dako) to detect macrophages. After incubation with poly-horseradish peroxidase-conjugated anti-rabbit BrightVision and poly-alkaline phosphatase-conjugated anti-mouse Bright Vision, (Immunologic), T-bet was visualized with NovaRed and macrophages with Vector Blue (Vector Laboratories), respectively.

### Identification and quantification of ILCs and other cell types

Whole slide images of the quadruple stained sections were registered in the user interface of the Philips image management system. In each stack of images, a region of interest of approximately 3 mm^2^ encompassing the area with the highest density of inflammatory cells (easily recognized in HE stained sections) was selected, annotated, and used for quantification. ILC1, ILC2, and ILC3 subsets were identified in these regions by the expression of T-bet, GATA3 and RORγt, respectively, but lacking lineage markers CD3, CD56, CD20 and CD79α. Subpopulations of T cells (T1, T2 and T17 cells) could be identified by the same TFs in cells that co-expressed CD3, but were negative for the other lineage markers. Cells that co-expressed CD3 and CD56 were considered NKT cells.

The numbers of ILC1s, ILC2s, ILC3s, T1, T2 and T17 cells, NK cells, NKT cells, B cells/plasma cells and the total number of lymphoid cells (all aforementioned cell types together) were counted and expressed as number of cells per mm^2^ and as percentage of the total number of lymphoid cells. The quantification was performed by two investigators. Discordance in interpretation of staining positivity was mediated through consensus, ensuring a 10% or less difference in the cell counting.

### Data analysis

Statistical analysis was performed with SPSS Statistics 23.0. (IBM, Armonk, New York, United States). First, a normality test was performed with Shapiro Wilk, followed by a non-parametric test. The differences in number (cells/mm^2^) and proportion (%) of ILC and T-cell subsets in relation to plaque development were compared using the Kruskal Wallis test. The differences between the ILC subsets and T-cell subsets in each plaque developmental stage were analyzed using the Friedman test. When the Kruskal Wallis test results were significant (*p* < 0.05), *post-hoc* correction for pairwise comparisons was performed with the Benjamini-Hochberg procedure.

## Results

### ILC1 is the dominant ILC subtype in human atherosclerotic plaques

**Figure 1:**
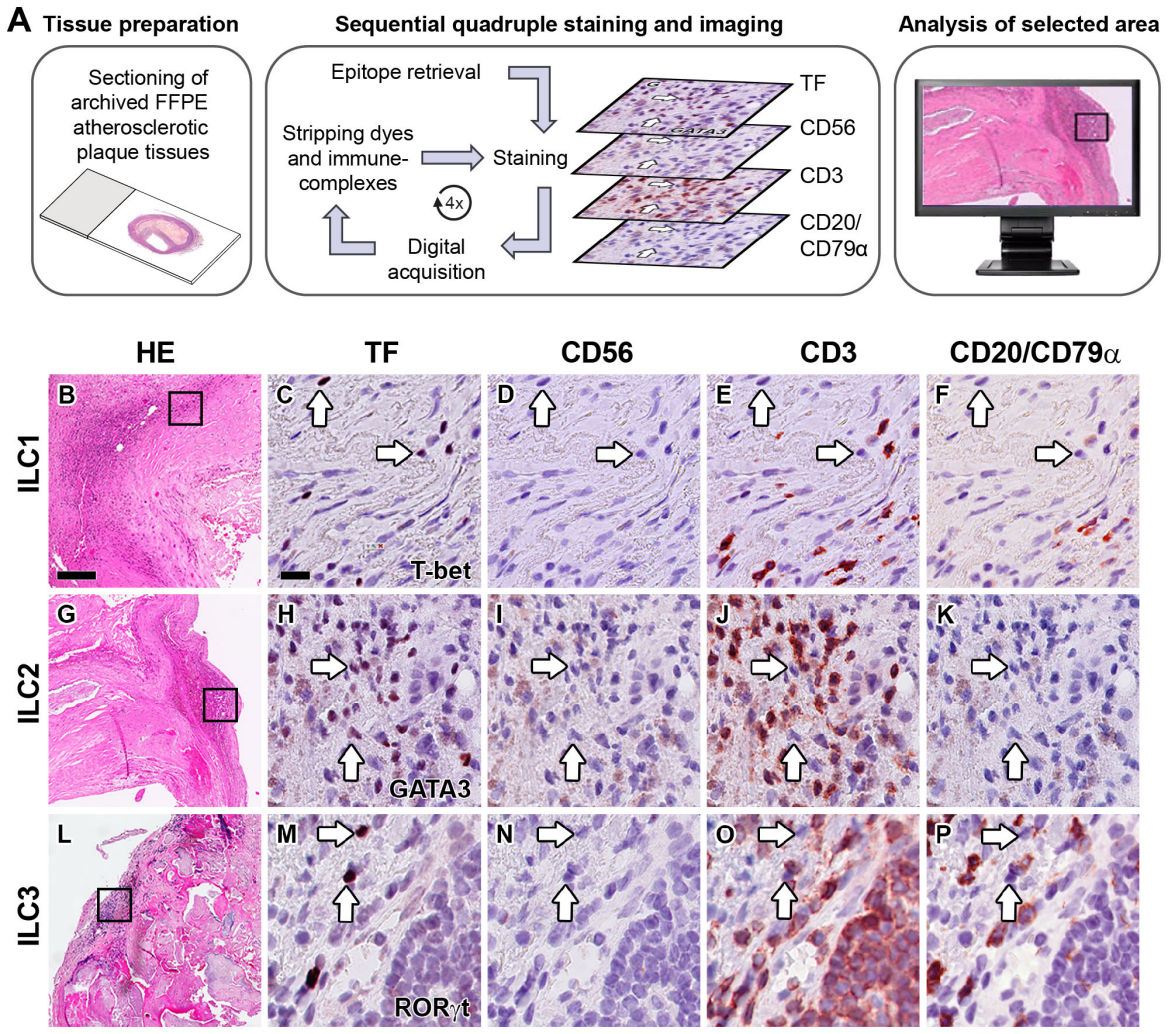
Identification of ILC1s, ILC2s and ILC3s in the intima of human atherosclerotic lesions. **(A)** Workflow of quadruple staining. Hematoxylin-eosin (HE) overview **(B, G, L)** and detailed (immunohistochemical) staining of carotid atherosclerotic plaques showing the identification of ILC1s **(C–F)**, ILC2s **(H–K)** and ILC3s **(M–P)**. Sections were stained with either T-bet **(C)**, GATA3 **(H)** or RORγt **(M)**, and subsequently digitized and destained. This workflow was repeated for CD56 **(D, I, N),** CD3 **(E, J, O)** and CD20/CD79α **(F, K, P)**. ILCs were identified by positive staining with the transcription factor (TF), but being negative for lineage markers CD56, CD3 and CD20/CD79α. Arrows indicate ILCs in sequentially stained sections. Scale bar in HE overview **(B)**: 200 µm. Scale bar in high power detail **(C)**: 25 µm. TF, transcription factor.

**Figure 2:**
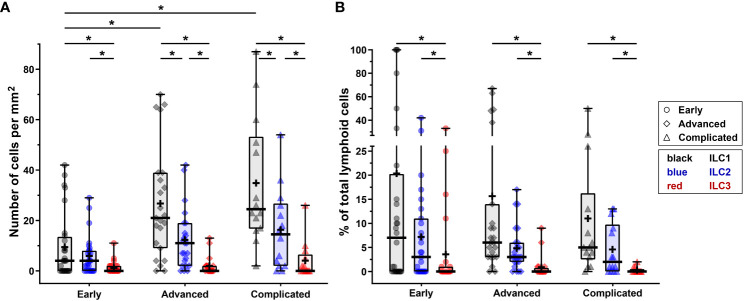
Quantification of ILC1s, ILC2s and ILC3s in different stages of atherosclerotic plaque development. **(A)** Numbers of ILC subsets (cells/mm^2^) present in the different stages (early, advanced, or complicated) of atherosclerotic plaques. **(B)** Proportion of ILC1s, ILC2s and ILC3s present in each plaque category, being expressed as a percentage of the total number of lymphoid cells. Data are expressed as box and whisker plots, with the median indicated by a bold horizontal line and the mean indicated by a **+** symbol. ILC2 and ILC3 are depicted in black, blue, and red, respectively, whereas the different stages are indicated by dots (early), diamonds (advanced), and triangles (complicated). (*p < 0.05).

In order to determine whether ILCs are present in early, advanced, and complicated human atherosclerotic plaques, we applied a multiplex quadruple staining protocol (25), using signature TFs to distinguish the ILC subsets (9, 11) and lineage markers CD3, CD56, CD20/CD79α to exclude T cells, NK cells, NKT cells, B cells and plasma cells (**Figure 1A**). We identified ILC1s (T-bet^+^Lineage^–^) in varying numbers in the vast majority (80%) of all specimens (**Figures 1B–F**), and interestingly, the numbers of ILC1s were significantly increased in advanced and even more in complicated plaques when compared to early lesions (**Figure 2A**). GATA3^+^Lineage^–^ ILC2s were also encountered in the majority (78%) of the lesions (**Figures 1G–K**), and like the ILC1s, their numbers were increased in advanced and complicated plaques when compared with early lesions, however, these differences were not statistically significant (**Figure 2A**). The RORγt^+^ Lineage^–^ ILC3s were found in 43% of the total plaques (**Figures 1L–P**) and their numbers did not significantly differ between early, advanced, and complicated lesions (**Figure 2A**).

**Figure 3:**
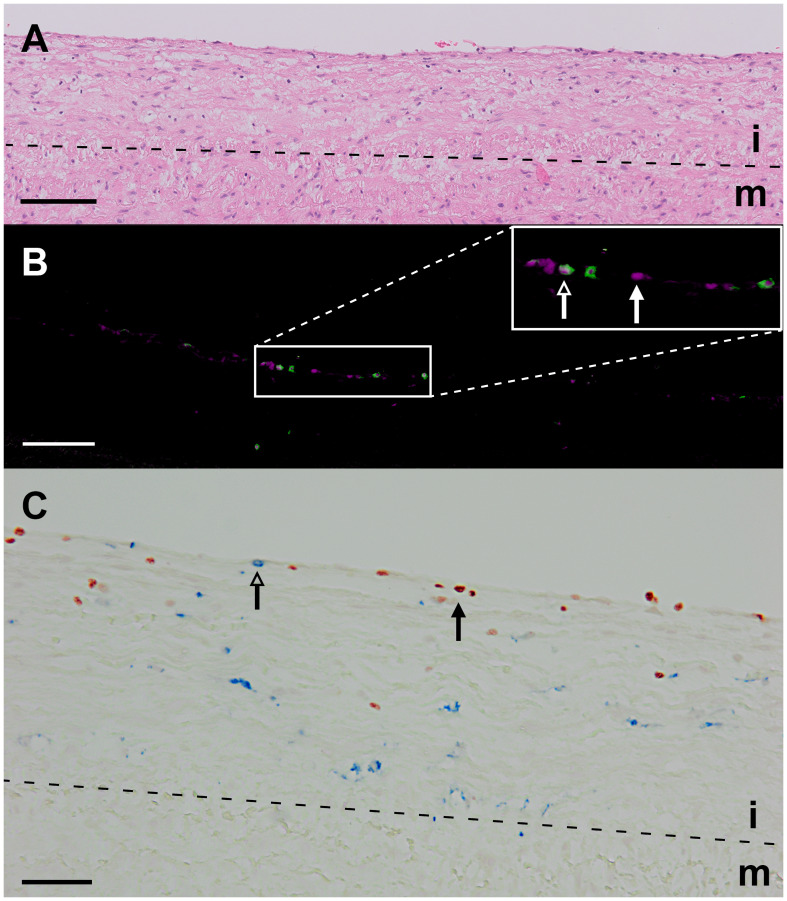
Identification of ILC1s in an early atherosclerotic plaque (fatty streak). **(A)** Hematoxylin-eosin staining. **(B)** False color multiplex staining showing T-bet in cyan and CD3 in green (closed and open arrowheads, respectively). This section was also stained for NK cells (CD56) and B cells (CD20/CD79α), but these cells were not present in this high power field. **(C)** Immunohistochemical double staining for T-bet (in blue, open arrowhead) and CD68 (red, closed arrowhead) showing that intralesional macrophages do not express T-bet. Scale bar equals 100 µm in **(A, B)** and 50 µm in **(C)**. i, intima; m, media.

In all stages of plaque development, the ILC1s were the most abundant ILC subset being significantly higher compared to ILC3s (**Figure 2A**). In the advanced and complicated plaque stages, the amount of ILC1s was also significantly higher than ILC2s, whose numbers, in turn, were also significantly higher than ILC3s (**Figure 2A**). All ILC subsets infiltrated in the intima showed a scattered distribution, with no special preferred location, except for a minor part of the ILC1s that were typically located along the innermost intima in close contact with the bloodstream (**Figures 3A, B**). Altogether, here we report the unprecedented discovery that ILCs are present in all developmental stages of human atherosclerotic plaques *in situ* and that ILC1 is the dominant ILC subtype.

### T-bet, GATA3 and RORγt expression by intraplaque non-ILC lymphocytes

**Figure 4:**
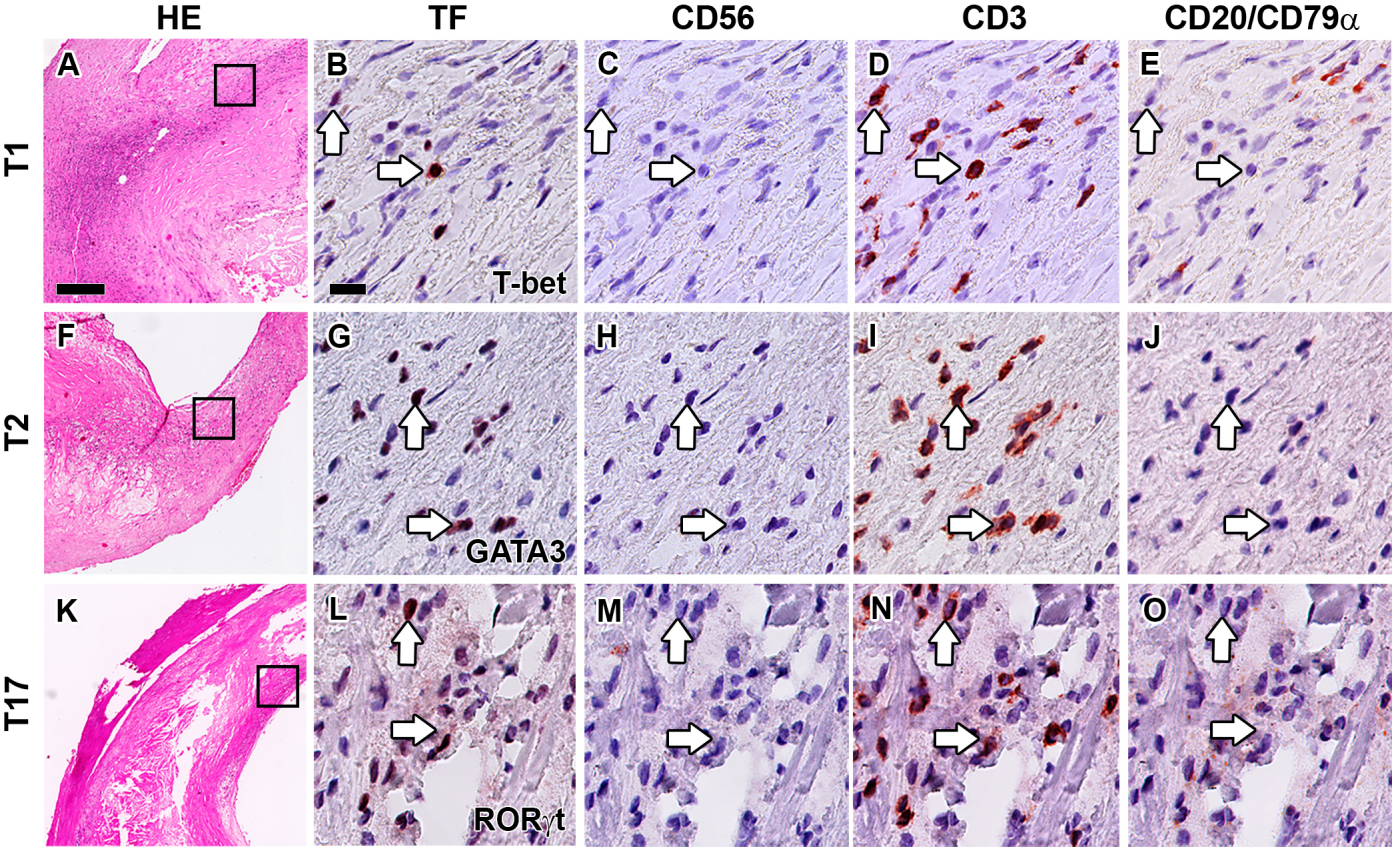
Identification of T1, T2 and T17 cells in the intima of human atherosclerotic lesions. Hematoxylin-eosin (HE) overview **(A, F, K)** and detailed staining of carotid atherosclerotic plaques showing the identification of T1 **(B–E)**, T2 **(G–J)** and T17 **(L–O)** cells. Sections were stained with either T-bet **(B)**, GATA3 **(G)** or RORγt **(L)**, and subsequently digitized and destained. This procedure was repeated for CD56 **(C, H, M)**, CD3 **(D, I, N)** and CD20/CD79α **(E, J, O)**. T1, T2 and T17 cells were identified by positive staining with TF and CD3, but being negative for CD56 and CD20/CD79α. Arrows indicate transcription factor (TF) positive T cells. Scale bar in HE overview **(A)**: 200 µm. Scale bar in high power detail **(B)**: 25 µm.

**Figure 5:**
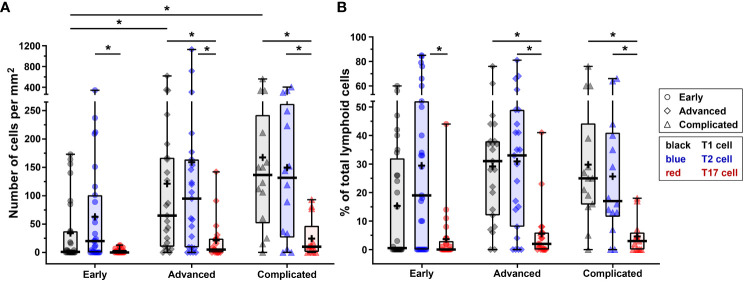
Quantification of T1, T2 and T17 cells in different stages of atherosclerotic plaque development. **(A)** Numbers of T1, T2 and T17 cells (cells/mm^2^) present in the different stages (early, advanced, or complicated) of atherosclerotic plaques. **(B)** Proportion of T1, T2 and T17 cells present in each plaque category, being expressed as a percentage of the total number of lymphoid cells. Data are expressed as box and whisker plots, with the median indicated by a bold horizontal line and the mean indicated by a **+** symbol. T1, T2 and T17 cells are depicted in black, blue, and red, respectively, whereas the different stages are indicated by dots (early), diamonds (advanced), and triangles (complicated). (*p < 0.05).

T-bet, GATA3, and RORγt are not only expressed by CD3^–^CD56^–^CD20/CD79α^–^ ILCs, but also by CD3^+^CD56^–^CD20/CD79α^–^ T1, T2, and T17 cells, respectively. A practical benefit of our multiplex quadruple staining method is that CD3 is applied as a separate color, enabling the concomitant determination of a particular ILC subset and its T cell subset counterpart within one tissue section. T1 cells (T-bet^+^CD3^+^CD56^–^CD20/CD79α^–^) were found in the majority of all lesions (74%) and their numbers were significantly increased in advanced and complicated plaques compared to early lesions (**Figures 4A–E**, **5A**). T2 cells (GATA3^+^CD3^+^CD56^–^CD20/CD79α^–^) were encountered in 77% of all specimens while no significant differences in numbers were noticed between different stages of plaque development (**Figures 4F–J**, **5A**). T17 cells (RORγt^+^CD3^+^CD56^–^CD20/CD79α^–^) were observed only in 60% of atherosclerotic lesions, and when compared to early lesions, their numbers showed a minor (not significant) increase in advanced and complicated plaques (**Figures 4K–O**, **5A**).

The numbers of T1 cells did not significantly differ from the amount of T2 cells in any stage of plaque development, but when compared to T17 cells, significantly higher numbers of T1 cells were present in advanced and complicated lesions (**Figure 5A**). The T2 cells significantly outnumbered T17 cells in all stages (**Figure 5A**).

**Figure 6:**
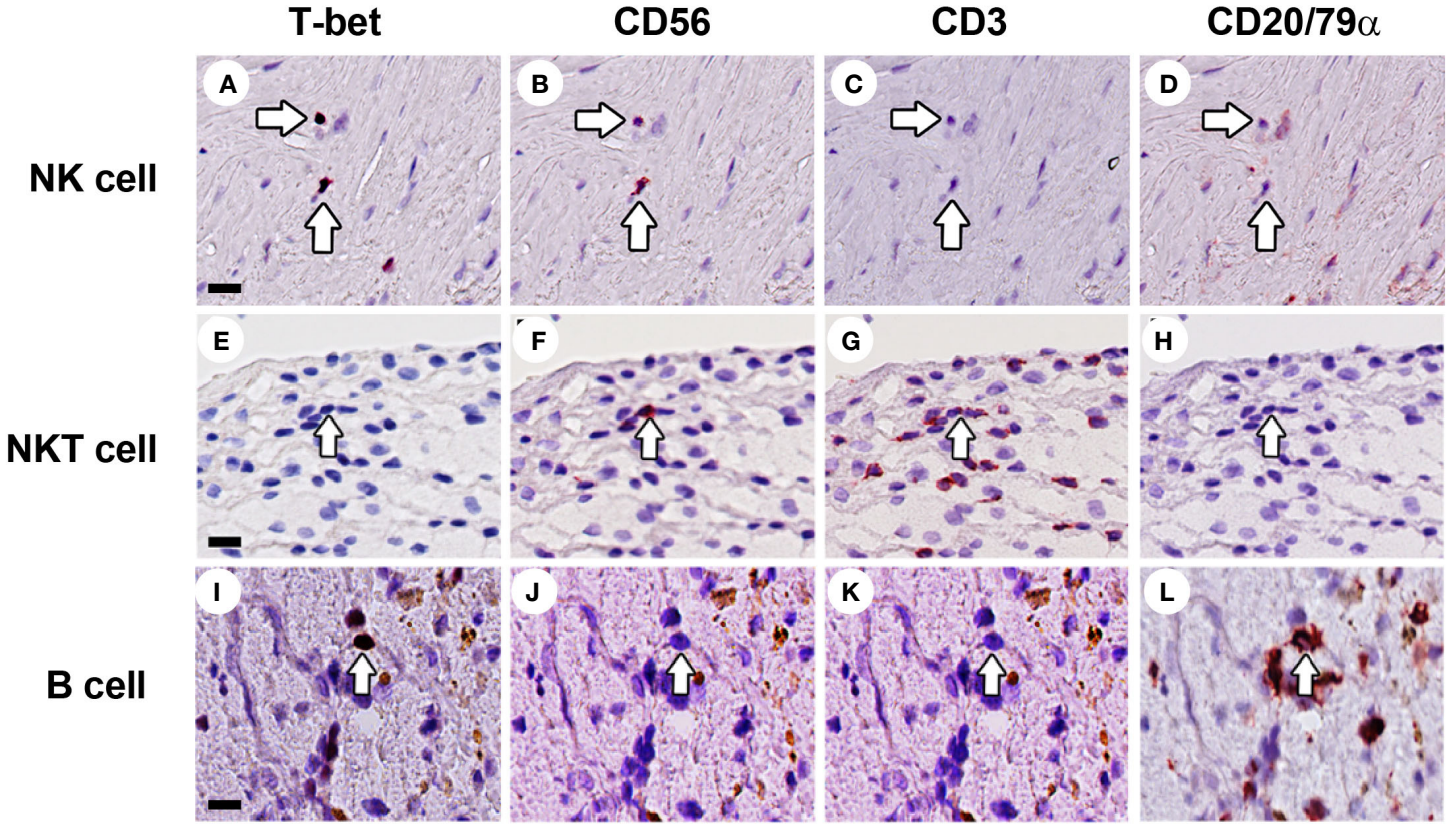
Identification of NK cells, NKT cells and B cells in human atherosclerotic lesions. Sections of carotid atherosclerotic plaques were sequentially stained with T-bet, CD56, CD3, and CD20/CD79α. **(A–D)** NK cells were identified as CD3^–^CD56^+^CD20/CD79α^–^ cells (arrow). **(E–H)** NKT cells were identified as CD3^+^CD56^+^CD20/CD79α^–^ cells (arrow). **(I–L)** B cells were identified as CD3^–^CD56^–^CD20/CD79α^+^ cells. Scale bar in **(A)**: 50 µm.

**Figure 7:**
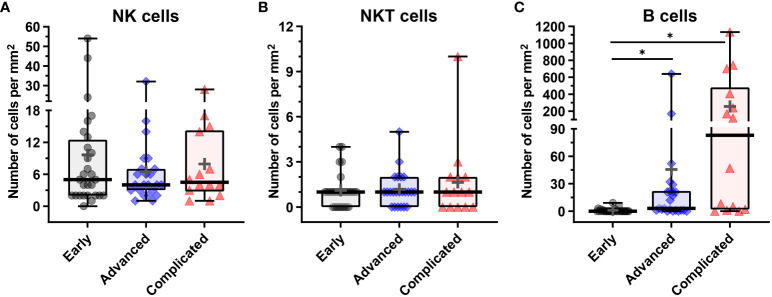
Quantification of NK, NKT and B cells in human atherosclerotic plaques. **(A)** Number of CD3^–^CD56^+^CD20/CD79α^–^ NK cells, **(B)** number of CD3^+^CD56^+^CD20/CD79α^–^ NKT cells, and **(C)** CD3^–^CD56^–^CD20/CD79α^+^ B cells present in early, advanced, or complicated atherosclerotic plaques. Data are expressed as box and whisker plots, with the median indicated by a bold horizontal line and the mean indicated by a **+** symbol. Numbers are expressed as cells/mm^2^ (*p < 0.05).

**Figure 8:**
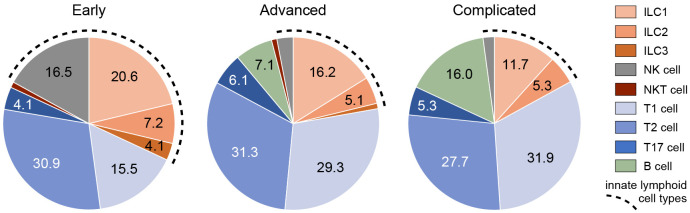
Frequencies of ILCs and other types of lymphoid cells in different stages of atherosclerosis. The proportion (means) of different types of innate and adaptive lymphocytes present in early, advanced, or complicated atherosclerotic plaques. The fragmented line indicates the total proportion of innate lymphocytes.

Although NK cells (CD3^–^CD56^+^CD20/CD79α^–^) were low abundant in advanced and complicated plaques (3.0% and 2.1% of the total lymphoid cells, respectively), the CD56^+^ NK cells represented a substantial proportion (16.5%) of the lymphoid cells in early lesions (**Figures 6A–D**, **7**, **8**). Less than 50% of the NK cells expressed T-bet. Identification of NK cells in atherosclerotic plaques is in concordance with data in the literature (27). NKT cells being double positive for CD56 and CD3 could only occasionally be detected: 1.0%, 0.6% and 0.3% of the total lymphoid cells in early, advanced and complicated plaques, respectively (**Figures 6E–H**, **7**, **8**). Remarkably, the vast majority of the NKT cells expressed GATA3.

CD20/CD79α^+^ B cells could not be detected in early plaques in the regions of interest that were used for quantification, and could only occasionally be encountered when inspecting the entire plaque. This is in line with a study by Munro et al. who were not able to detect B cells in the intima of human aortic fatty streaks (28). In the advanced and complicated atherosclerotic plaques, we found substantial numbers of B cells: 7.1%, 16% of the total lymphoid cells, respectively (**Figures 6I–L**, **7**, **8**). These intraplaque B cells in the intima did not show any structural organization, as has earlier been demonstrated for adventitial B cell infiltrates (29), but were solitary cells with a scattered distribution. T-bet was occasionally found in cells that co-expressed CD20/CD79a, a phenotype earlier described in the literature as age-associated B cells (30).

Given that IFN-γ-secreting Th1 cells are the most prominent T cell subtype in atherosclerotic plaques (2), that IFN-γ may induce T-bet in myeloid cells (31) and that macrophages are highly abundant in these plaques (2), we examined whether CD68^+^ intraplaque macrophages showed T-bet expression. We found that the expression of T-bet and CD68 was mutually exclusive (**Figure 3C**), supporting our strategy to use T-bet in combination with lineage markers for detection of ILC1s in tissue sections.

### ILC1s comprise a substantial proportion of the lymphocytes in early atherosclerotic plaques

To put the presence of ILCs in atherosclerotic plaques in physiologic perspective, we expressed their frequency as a proportion of the total lymphoid population: i.e. all ILCs, T cells, NK cells, NKT cells, and B cells together. Interestingly, our data suggest that the amount of ILC1s is relatively high in early atherosclerotic lesions. ILC1s comprised more than 10% of the lymphocytic infiltrates in half of the early lesions; in 5 out of the 28 early cases the proportion of ILC1s was even ≥ 50%, and unexpectedly, 3 of these 5 specimens only comprised of ILC1s in absence of conventional lymphocytes (**Figure 2B**, **8**). The *in vivo* enrichment of ILC1s in the early stage of atherosclerosis is immunohistochemically illustrated in **Figures 3A, B**, showing an aortic fatty streak in which approximately equal amounts of T-bet^+^CD3^–^ ILC1s and T-bet^+^CD3^+^ T cells are present.

In a quarter of the early plaques, the ILC2s comprised more than 10% of the lymphocytic population, whereas this was found for ILC3s in only 4 cases (**Figures 2B**, **8**). In the more advanced plaque stages, the ILC1 fraction was also found to be more frequent than ILC2s (significant in the advanced stage) and ILC3s (significant in all stages) (**Figures 2B**, **8**). ILC3 was the least frequent ILC subset in all stages of plaque development (**Figures 2B**, **8**). Overall, we found that ILC1s made up a substantial part of the total lymphoid population in atherosclerotic plaques, in particular in the early stage. Although the numbers of ILCs showed a rise in the course of plaque advancement, when expressed as frequency of total lymphocytic cells, the proportion of all three ILC subsets gradually decreased along with the progress of plaque development due to the more progressive increment of intraplaque T cells and B cells.

When expressed as a percentage of the total number of lymphoid cells, T1 and T2 cells always significantly outnumbered T17 cells, but no significant differences were observed between T1 and T2 cells (**Figures 5B**, **8**). All in all, T17 cells were clearly the least frequently occurring T cells subset in all stages of plaque development. When we compared the frequencies of ILCs with their respective T-cell subset counterparts, it appeared that the conventional T cell subsets significantly outnumbered their ILC counterparts in all stages, with two exceptions (**Figure 8**). Firstly, interestingly and unexpectedly, the proportion of ILC1s appeared to be higher than T1 cells in early lesions, although this difference was not statistically significant. Secondly, the proportions of ILC3s and T17 cells appeared to be similar in early lesions although both were present in relatively low amounts.

Of note, ILC1s and NK cells, both forming group 1 ILC, comprised almost 40% of the lymphocytic infiltrates in the intima in early plaques, and interestingly, all innate cell types together even comprised up to 50% of the intraplaque lymphocytes (**Figure 8**), suggesting involvement of the innate immune system in the early stage of atherogenesis. In the course of plaque development, the proportion of ILCs gradually reduced to less than 20% in complicated plaques.

## Discussion

ILCs have been implicated in the pathogenesis of a broad spectrum of diseases. Here, we report the previously unappreciated presence of ILC1s, ILC2s, and ILC3s in the early, advanced, and complicated stages of human atherosclerosis *in situ*. The identification of the distinct ILC subsets was based on the differential expression of hallmark TFs T-bet, GATA3 and RORγt (present on ILC1s, ILC2s, and ILC3s, respectively) and the absence of lineage markers CD3, CD56, CD20 and CD79α. We demonstrate that ILCs are greatly enriched in atherosclerotic lesions when compared to the frequency of ILCs in peripheral blood, which has been determined by flow cytometry to be less than 0.1% of the leukocytes in circulation of healthy volunteers (15, 16). It is known that the frequencies of leukocyte-subsets in the peripheral blood shift as a result of acute coronary syndromes (32). Unfortunately, we do not know the frequency of ILCs in the blood circulation of the patients included in this study. A detailed analysis of ILC subsets in the peripheral blood of patients may help to understand a possible role for ILCs in atherosclerotic disease. In this respect, it is interesting to note that there is a shift of ILC1 and ILC2 levels in the peripheral blood after cerebral infarction, which increased and decreased, respectively (33). Based on our current data, we cannot conclude whether the intraplaque enrichment of ILCs were the result of active recruitment and homing of circulating ILCs into the atherosclerotic tissue or due to local proliferation and/or polarization.

In all stages of atherosclerosis, ILC1s were found to be the most abundant ILC subset. Moreover, ILC1s made up a considerable part of the total lymphoid population in atherosclerotic tissue, in particular in early lesions where ILC1s outnumbered the number of conventional lymphocytes in many samples, and intriguingly, in three cases they were found to be the only lymphoid cell type present in the early plaque. When we compared the abundance of ILC subsets with those of T cell subtypes, we found that in all stages of atherosclerosis, each ILC subset is significantly outnumbered by its conventional T lymphocyte counterpart, except for ILC1s in early lesions. This large quantity of ILC1s in the early stages of atherosclerosis suggests that they may play a role in the pathogenesis of these lesions. As ILC1s are still present in relatively large amounts in later stages of atherosclerosis, they may be important players in the maintenance of the inflammatory response in the plaque as well. Of course, solely based on immunohistochemical data, it is difficult to predict to what extent ILCs contribute to the local immune responses in atherosclerotic plaques *in vivo*. However, given that ILCs are important regulators of immunity as they can directly produce effector cytokines in response to subset-specific, local tissue-derived factors (without requirement for antigen-specific activation) and their remarkable local enrichment hint that they are not innocent bystanders and likely participate in the pathogenesis of atherosclerosis. This hypothesis is supported by a recent study with an experimental mouse model of atherosclerosis, showing that ILC1s aggravate atherosclerosis (20).

Atherosclerosis is often considered as a Th1 cell-mediated chronic inflammatory disease (3, 34). Type-1 cytokines (TNF-α, IFN-γ) are known to aggravate atherosclerosis in experimental models (1, 2, 20, 35). In the literature, Th1 cells were found to be the predominant subset among plaque-infiltrating lymphocytes in carotid and femoral endarterectomies, based on immunohistochemical analysis of cytoplasmic cytokine protein and RT–PCR analysis of cytokine transcripts in plaque RNA (36). Indeed, Th1 cells have been identified in human aortic plaques, using double-staining for CD4/T-bet to identify (CD4^+^T-bet^+^)-Th1 cells and (CD4^+^T-bet^–^)-non-Th1 cells in aortic specimens. However, the authors concluded that Th1 cells were not the dominant Th lineage cell found in the different atherosclerotic stages (37). In our quadruple staining approach, we did not include antibodies against CD4 and CD8, and therefore we cannot further differentiate the helper and cytotoxic T-cell populations into Th1/Th2/Th17 or Tc1/Tc2/Tc17 subsets, respectively. Our results indicate that both T1 and T2 cells dominate in all stages of atherosclerosis, while not being significantly different.

ILC2s were identified in all stages of human plaque development and generally were the second major ILC type after the ILC1s. The type 2 cytokines (IL-4, IL-5, IL-10, IL-13) produced by ILC2s typically antagonize type 1 responses, and are therefore often regarded as atheroprotective (38). In an experimental model, it was shown that ILC2s indeed are able to inhibit the development of atherosclerotic plaques in mice (21, 22, 24). As ILC1s outnumber ILC2s in atherosclerotic plaques, it can be reasoned that the intraplaque ILC2s are unable to counterbalance the atherogenic activity of the neighboring ILC1s. On the other hand, we have demonstrated that large numbers of T2 cells are present in the plaques potentially able to produce the atheroprotective type 2 cytokines. Still, the role of Th2 cells (and thus type-2 immunity) in atherosclerotic disease remains controversial (39).

Both ILC3s and T17 cells are the least lymphoid subsets in human atherosclerosis. Any role for ILC3s in atherosclerosis is unknown and the contribution of T17 cells still remains inconclusive. Th17-derived IL-17 has been detected in human atherosclerotic lesions (40) and is suggested to play a potential proatherogenic role in plaque instability (41). On the other hand, another study on carotid plaques showed that IL-17 associates with vascular smooth muscle cells producing collagen and thus supports the role of Th17 in plaque stability (42).

In recent years, several mass cytometry (CyTOF) and single-cell RNA sequencing (scRNA-seq) analyses have been performed of human atherosclerotic plaques obtained from patients undergoing carotid endarterectomy (7, 43–47), but surprisingly however, none of these high-dimensional single-cell studies have described the presence of ILCs. There are several reasons to explain why ILCs were not identified in these studies. Firstly, all studies have in common that they focused on fine-tuning the subsets of the major populations (T cells, macrophages and vascular smooth muscle cells) residing in atherosclerotic plaques concomitantly ignoring the undefined minor populations, which comprised up to 10% of the plaque infiltrating leukocytes and probably may have included ILCs. This notion is stressed by a recent report in which the scRNA-seq data of Wirka et al. (44) were re-analyzed and an ILC population was discovered (48). Secondly, ILCs lack specific markers hampering identification of these cells across healthy and inflamed tissue types. Moreover, phenotypical discrepancies between ILCs of different tissue origin (49) further complicate identification of these cells. Nevertheless, the hallmark TFs that dictate the functionality of the three distinct ILC subsets still can be used to accurately recognize ILCs across different tissue types. Thirdly, transcriptomic analysis has demonstrated gene-expression overlap between FACS-purified ILC1s and NK cells (50), and furthermore, ILC1s express transcripts that encode variable regions of the T cell receptor and several other T cell specific markers (51). Because of the lack of specific markers and partial overlap with T cells and NK cells, the minor populations of ILC subsets may be invisible as separate clusters in the scRNA-seq studies. Fourthly, detection of a particular cell type by IHC does not necessarily mean that these cells should also be detected by high-dimensional single-cell techniques such as CyTOF and scRNA-seq. For example, IHC revealed an abundant presence of Th1 cells in atherosclerotic lesions (36), but remarkably, they do not appear as an identifiable cluster by scRNA-seq (52). The presence of NK cells in human atherosclerotic plaques was shown by IHC (27), but strikingly, a separate NK cell cluster was observed only in some scRNA-seq studies (7, 44, 47), whereas in other studies such cluster was absent (43, 46). Furthermore, high numbers of CD11b^+^ myeloid cells can be observed in plaques by IHC, but these cells appeared only as a minor cluster using CyTOF (53). Fifthly, a recent comprehensive meta-analysis (52) of the leukocyte infiltrate in atherosclerotic plaques in mice demonstrated the presence of ILC2s in two out of nine included scRNA-seq studies (53, 54) as well as in one out of two CyTOF studies (55), indicating large variability between these types of studies. Lastly, in contrast to all scRNA-seq studies, in which only carotid endarterectomy specimens (representing a late stage of atherosclerosis) were investigated, we studied different stages of atherosclerosis and found abundant enrichment of ILC1s in particular in early plaques, whereas in later stages the relative frequency was reduced because of a strong increment of T cells and B cells.

Our data rely solely on a single method, posing a possible limitation. Hence, conducting further experiments using alternative methods and techniques is imperative to validate our findings. Additionally, it’s important to note that ILC- and lymphocyte-subset frequencies were determined in plaque areas with the highest inflammatory activity, potentially limiting their representativeness of the entire atherosclerotic plaque.

In conclusion, we have identified the presence of all three subsets of ILCs in all developmental stages of human atherosclerotic disease, ILC1s being the most abundant. As ILCs have not been detected before in human atherosclerotic plaques they represent novel players in atherogenesis in man. Considering that ILCs are potent immunomodulatory cells, our data suggests involvement of ILCs in the initiation and progress of atherosclerotic disease and may represent an important alternative target to treat or prevent atherosclerosis. However, given the complicated regulation of the (chronic) inflammatory process in atherosclerosis, as well as ILC biology, more research is needed to fully understand their precise role in the pathogenesis of atherosclerosis and other cardiovascular diseases.

## Data availability statement

The original contributions presented in the study are included in the article/supplementary material. Further inquiries can be directed to the corresponding author.

## Ethics statement

The studies involving humans were approved by Medisch Ethische Toetsingscommissie Amsterdam University Medical Centers, location AMC Meibergdreef 9 1105 AZ Amsterdam The Netherlands. The studies were conducted in accordance with the local legislation and institutional requirements. The ethics committee/institutional review board waived the requirement of written informed consent for participation from the participants or the participants’ legal guardians/next of kin because All specimens were leftover materials from clinical interventional procedures and were anonymously used.

## Author contributions

KP: Conceptualization, Data curation, Formal analysis, Investigation, Visualization, Writing – original draft, Writing – review & editing, Validation, Project administration. MT: Conceptualization, Data curation, Formal analysis, Methodology, Visualization, Writing – original draft, Writing – review & editing, Investigation. GK: Conceptualization, Investigation, Writing – review & editing. MW: Writing – review & editing, Resources. LH: Writing – review & editing, Resources. CP: Conceptualization, Investigation, Writing – review & editing. AW: Conceptualization, Formal analysis, Writing – review & editing, Investigation. OB: Conceptualization, Formal analysis, Investigation, Supervision, Writing – original draft, Writing – review & editing, Methodology, Visualization.
